# Genome-wide association testing in malaria studies in the presence of overdominance

**DOI:** 10.1186/s12936-023-04533-2

**Published:** 2023-04-10

**Authors:** Morine Akoth, John Odhiambo, Bernard Omolo

**Affiliations:** 1 Strathmore Institute of Mathematical Sciences, Strathmore University, Ole Sangale Road, Nairobi, Kenya; 2 Division of Mathematics & Computer Science, University of South Carolina-Upstate, 800 University Way, Spartanburg, USA; 3 School of Public Health, Faculty of Health Science, University of the Witwatersrand, Johannesburg, South Africa

**Keywords:** Allelic test, Case–control study, Genome-wide association, Malaria, MAX test

## Abstract

**Background:**

In human genetics, heterozygote advantage (heterosis) has been detected in studies that focused on specific genes but not in genome-wide association studies (GWAS). For example, heterosis is believed to confer resistance to certain strains of malaria in patients heterozygous for the sickle-cell gene, haemoglobin S (HbS). Yet the power of allelic tests can be substantially diminished by heterosis. Since GWAS (and haplotype-associations) also utilize allelic tests, it is unclear to what degree GWAS could underachieve because heterosis is ignored.

**Methods:**

In this study, a two-step approach to genetic association testing in malaria studies in a GWAS setting that may enhance the power of the tests was proposed, by identifying the underlying genetic model first before applying the association tests. Generalized linear models for dominant, recessive, additive, and heterotic effects were fitted and model selection was performed. This was achieved via tests of significance using the MAX and allelic tests, noting the minimum *p*-values across all the models and the proportion of tests that a given genetic model was deemed the best. An example dataset, based on 17 SNPs, from a robust genetic association study and simulated genotype datasets, were used to illustrate the method. Case–control genotype data on malaria from Kenya and Gambia were used for validation.

**Results and conclusion:**

Results showed that the allelic test returned some false negatives under the heterosis model, suggesting reduced power in testing genetic association. Disparities were observed for some chromosomes in the Kenyan and Gambian datasets, including the sex chromosomes. Thus, GWAS and haplotype associations should be treated with caution, unless the underlying genetic model had been determined.

**Supplementary Information:**

The online version contains supplementary material available at 10.1186/s12936-023-04533-2.

## Background

In human genetics, heterozygote advantage (heterosis) has been detected in studies that focused on specific genes [1, 2], but not in genome-wide association studies (GWAS). For example, heterosis is believed to confer resistance to certain strains of malaria in patients heterozygous for the sickle-cell gene haemoglobin S (HbS). Yet the power of allelic tests can be substantially diminished by heterosis [3]. Since GWAS (and haplotype associations) also utilize allelic tests [4, 5], it is unclear to what degree GWAS could underachieve because heterosis is ignored. GWAS has been conducted by testing many genetic variants to find a statistical association with a disease or a particular trait. Steps for conducting GWAS include data collection for the selected study population, genotyping, data processing, and testing for association [6].

Simulation studies by Omolo and colleagues [3] showed that allelic tests underperform in the presence of heterosis, a condition found in some diseases such as malaria and sickle cell anaemia [1]. It is unclear how the allelic tests conducted at millions of single nucleotide polymorphisms (SNPs) would perform under heterotic conditions.

Existing tests for association studies include Pearson’s chi-square test, the allelic test, the Cochran Armitage trend tests, and the MAX test among other tests. Pearson’s Chi-square test and the Cochran Armitage trend test (CATT) [7, 8] are known for genetic association using case–control samples. The trend tests corresponding to the three commonly used genetic models are the dominant, recessive, and additive [7, 8]. The MAX test was proposed by Loley et al. [9], Gonzalez et  al. [10], Zhang et  al. [11], and Horthon et  al. [12]. The test allowed for the underlying genetic model to be selected as opposed to assuming a particular genetic model [7]. Zhang et  al. [11] developed an algorithm to calculate empirical and asymptotic *p*-values for the MAX and allelic tests. The algorithm has reduced the computation burden of association tests. Zintzaras et  al. [13] studied the degree of dominance which attempted to include the heterotic situation on a continuous scale. The simulation study showed that the method was promising for model selection. Gonzalez et  al. [10] derived the asymptotic form of the MAX test and estimated its significance level based on the three genetic models. Similar to the tests developed by Zang et al. [11], the test showed reduced computational burden. However, an extension of the heterosis situation would be important for some traits. Horthon et  al. [12] used conditional reference distribution for the MAX test in three dimensions and showed that it is asymptotically normally-distributed with estimated parameters [14]. Similar to Horthon et  al. [12], the main interest lies in genome-wide association testing with heterosis being one of the genetic models. The existing tools for analysis have been extended in GWAS to include the heterotic model. See [15–17] for a detailed review of robust tests and their applications to genetic association studies.

In this study, a two-step approach to genetic association testing in malaria studies in a GWAS setting was proposed that may enhance the power of the tests by identifying the underlying genetic model before applying the association tests. Firstly, generalized linear models for the dominant, recessive, additive, and heterotic effects were fitted using case–control genotype data. The model selection was then performed using the MAX test procedure [12]. Here, the distribution of the MAX test was extended to cater to the heterotic effect in four-dimensional test statistics to yield the MAX4 test. The model with the smallest *p* value was selected for different markers. The *p*-values were adjusted for multiple comparisons using the Bonferroni method for SNPs with an allelic odds ratio greater than or equal to 1.5. The most significant SNPs were selected based on a threshold of $5 \times 10^{-8}$. Using the MAX4 and the allelic tests, statistics and p-values were estimated to determine SNPs significance across all the genetic models and perform the selection of the correct model. The estimated *p*-values of the MAX4 test were obtained using the parametric bootstrap (boot), bivariate normal (bvn), and the asymptotic method (asy) [11]. Genotype datasets were simulated under the Hardy-Weinberg equilibrium (HWE), assuming the multinomial distribution for cases and controls. The MAX4 and the allelic tests were performed on the simulated data sets to achieve model selection and to test for significance. An example dataset with 17 SNPs [11], and malaria genotype data from the Kenyan and Gambian populations with unrelated individuals were used for validation (https://www.ebi.ac.uk/ega/).

## Methods

### Genetic model

Consider a genetic marker with alleles *A* and *S* with genotypes *AA*, *AS*, and *SS* as shown in Table 1. The distribution of the genotypes from alleles *A* and *S* is found in Sasieni [8]. Assume *A* is the allele causing disease, which confers a high risk of malaria disease. The corresponding three genotypes are *AA*, *AS* and *SS*, denoted by $g_0=SS$, $g_1=AS$, and $g_2=AA$. The genotype frequencies $g_i=P(G_i)$ for $i=0,1,2$. The allele frequencies assume $P(A)=p$ and $P(S)=1-p=q$. HWE is assumed to hold, i.e. $g_0=q^2$, $g_1=2pq$, and $g_2=p^2$. The probability of being diseased given a particular genotype (penetrance), is given by $f_i=P(case|g_i)$ and the disease prevalence by $k=P(case)=\sum f_ig_i$, for $i=0,1,2$. Let the genotype counts of $g_0$, $g_1$ and $g_2$ in *r* cases and *s* controls be represented by $(r_0, r_1, r_2)$ and $(s_0,s_1,s_2)$ respectively, with $n_i=r_i+s_i$ where $i=0,1,2$ and $n=r+s$. Consider the penetrance relation among the different modes of inheritance. For the additive model, the penetrance relation is $f_0<f_1<f_2$, and the number of alleles raises the disease risk. For the dominant model, one *A* allele in the heterozygous phenotype is sufficient to cause a disease similar to two copies of the *A* allele, i.e *AA* genotype. The penetrance relationship is $f_0 <f_1\simeq f_2$. For the recessive model, the penetrance relationship is $f_0\simeq f_1< f_2$ and for the overdominant model (positive heterosis), the heterozygous genotype *AS* has the largest effect on disease risk, i.e $f_1> f_0,f_2$. Using the penetrance relation, we represent the overdominant situation for the MAX4 test using a score vector (0,1,0). The score vectors for dominant, recessive, and additive models are (0,1,1), (0,0,1), and (0,1,2) respectively. Table 2 shows the count of cases and controls by heterosis (overdominance). Define the genotype relative risk (GRR) as $f_i/f_0=\lambda _i$. Under different genetic models, a test for the null hypothesis $H_0:\lambda _i=1$ against the alternative $H_A:\lambda _i>1$ is performed.

### Simulated genotype data

Genotype data sets from a case–control study design were simulated. The frequency of both cases and controls maintained the HWE under multinomial distribution. Data were also simulated to violate the HWE assumption of allele frequencies $p^2$, 2*pq*, and $q^2$ for AA, AS, and SS genotypes, respectively. The allelic and the MAX4 tests were performed on different sample sizes. Using samples with 500 to 5,000 SNPs, genotype datasets were simulated using varying allele frequencies. Multinomial distribution was assumed for the cases and the controls. The initial probability of allele *A* was set at 0.1 and was used to determine the genotype distributions under the conditions of HWE [9]. A comparison of the allelic and the MAX4 test results was performed on the selected genetic models.

### Example dataset

The MAX4 test was applied to an example dataset (Additional file 1: Table S1) containing 17 common SNPs from age-related macular degeneration(AMD) [18], prostate cancer (PC) [19], breast cancer(BC) [20], and hypertension(HP) [21] studies and obtained significant results [11]. The *Rassoc* [11] package in *R* was used to generate the statistics and the *p*-values of the tests. This *R* package has Monte Carlo and asymptotic algorithms of the MAX3, CATT, allelic, and other commonly used tests in case–control studies. The algorithms calculated the *p*-values using the parametric bootstrap method, the bivariate normal distribution, and the asymptotic null distribution method. The algorithms were improved to incorporate the heterotic effect using the overdominance-related penetrance function.

### Malaria datasets

Malaria datasets with genotype data for cases and controls from two populations obtained from the Gambia and Kenya were used (https://www.ebi.ac.uk/ega/). There were 3340 samples from Kenya and 2780 samples from the Gambia in the datasets. Each sample had 23 chromosomes, including the sex chromosome. There were different frequencies of markers on each chromosome. All cases were diagnosed in a hospital, where blood samples from children diagnosed with severe malaria were collected. The controls were from within the general population and from new births with unrelated individuals. The blood samples were from the same geographic area as the cases. Deoxyribonucleic acid (DNA) was extracted from blood samples and examined at SNP Illumina arrays [22]. To process the data on the arrays, various sets of genomic calls were utilized. SNP allele names (A, C, T, G), identification numbers (ID), chromosomal positions, and SNP names were retrieved from input files. Other variables included sex, ethnicity, and country of origin.

The malaria datasets for the study were under EGA data *EGAS*00001000807 from Kenya and Gambia; dataset ID EGAD00010000570 (1544 controls and 1711 cases) for the Kenyan population and dataset ID EGAD00010000572 (1533 controls and 1247 cases) for the Gambian population. Different samples were picked from different geographical locations across the two countries to enhance genetic diversity in African countries. The initial study and description of the datasets are available at Band et al. [22]. *SNPTEST* *v*2.4.1 software was used to pre-process data to obtain case–control summary statistics on genotype counts, chromosome positions, allele frequency, and odds ratios (https://mathgen.stats.ox.ac.uk/genetics_software/snptest/snptest_v2.4.1.html). The MAX4 and the allelic tests were performed in the presence of an overdominant model. All statistical analyses were conducted in R studio version 4.2.0 [23].

### Cochran-Armitage trend test

The Cochran-Armitage trend test (CATT) and the chi-square have been well-studied for single variants [8]. The CATT is defined as

1 $$\begin{aligned} CATT=\frac{U}{(Var(U))^{1/2}} \end{aligned}$$

where

2 $$\begin{aligned} U=\frac{1}{n}\sum _{i=0}^{2}x_i(rs_i-sr_i) \end{aligned}$$

and

3 $$\begin{aligned} Var(U)=\frac{rs}{n}\left( \sum _{i=0}^{2}x_i^2n_i-\sum _{i=0}^{2} (x_in_i)^2\right) , \end{aligned}$$

where *r* is the number of cases, *s* is the number of controls and *n* is the total number of cases and controls. $n_i=r_i+s_i$, for $i=0,1,2$. $(x_0,x_1,x_2)$ represents the genotype score vectors for respective genotype models [24]. Consider the CATT of the form

4 $$\begin{aligned} Z_x=\frac{n^{0.5}\sum _{i=0}^2 x_i (sr_i-rs_i)}{[\frac{rs}{n^3}[n\sum _{i=0}^2 x_{i}^2 n_i-(\sum _{i=0}^2 x_in_i)^2]]^{0.5}} \end{aligned}$$

Under the overdominant model, with score vector (0,1,0) equation 4 becomes

5 $$\begin{aligned} CATT_{HET}= \frac{n^{0.5} x_1 (sr_1-rs_1)}{[\frac{rs}{n^3}[n x_{1}^2 n_1-( x_1n_1)^2]]^{0.5}} \end{aligned}$$.

**Table 1:** Count of cases and controls in the genotype model

	AA	AS	SS	Total
Cases	$r_{0}$	$r_{1}$	$r_{2}$	r
Control	$s_0$	$s_1$	$s_2$	s
Total	$n_0$	$n_1$	$n_2$	n

**Table 2:** Count of cases and controls in the heterosis model

	AA+SS	AS	Total
Cases	$r_0+r_2$	$r_1$	r
Control	$s_0+s_2$	$s_1$	s
Total	$n_0+n_2$	$n_1$	n

### The MAX test

The MAX test statistic is defined $Z_{max}=max(|Z_0|,|Z_{1/2}|,|Z_1|)$ [24]. It considers the three common genetic models. An extension of the test statistic to include an overdominant genetic model with a score vector (0,1,0) was proposed and denoted as the MAX4 test. The MAX4 statistic, $Z_{max4}$, was the maximum of the absolute CATT over four genetic models and it was defined as

6 $$\begin{aligned} Z_{max4}=max(|CATT_{ADD}|,|CATT_{DOM}|,|CATT_{REC}|,|CATT_{HET}|), \end{aligned}$$

where the genetic $CATT_{DOM}$, $CATT_{REC}$, $CATT_{ADD}$, and $CATT_{HET}$ were the CATTs under dominant, recessive, additive, and heterotic models respectively. The four test statistics asymptotically follow standard normal distribution *N*(0, 1) and can be expressed as a joint density function $f(z_1, z_2, z_3, z_4;\Sigma )$ where $\Sigma$ is the 4 by 4 variance-covariance matrix. Using integrate function in R, one can estimate probability under the curve for a given data hence p-value is obtained as follows

7 $$\begin{aligned} Pr(|Z_{max4}|<m)=\int _{-m}^m\int _{-m}^m\int _{-m}^m\int _{-m}^m f(z_1,z_2,z_3,z_4;\Sigma )dz \end{aligned}$$

Consider a case–control situation with proportions $p_0$, $p_1$, and $p_2$ for genotypes $g_0$, $g_1$ and $g_2$, respectively. The asymptotic means and variance for the multivariate normal distributions are used [25]. Therefore, the distribution of $Z_{MAX4}$ follows a four-variate normal distribution with asymptotic variance $p_i(1-p_i)$ and covariance $-p_ip_j$. Under no association, the test statistics have a mean vector of zero. Derivation of the correlation coefficients over three genetic models is discussed in [10, 11]. Parametric bootstrap in *m* replicates was used to approximate the null distribution of the MAX4. The *p*-values were estimated from the empirical null distribution of the MAX4 [11].

## Results

### Simulation study and example datasets

**Table 3:** The test statistics and the *p*-values of MAX4 and the allelic test procedures for the 17 SNPs reported in Additional file 2: Table S1 using the three approaches: the parametric bootstrap (boot), the bivariate normal approach (bvn) and the asymptotic approach (asy) for the case of the MAX4 procedure

SNPs	$P-values$ using different Methods						
	Statistic	MAXboot	MAXbvn	MAXasym	Alle-statistic	AllelicP-value	Model
rs380390	5.11	1.0E−06	2.0E−06	8.6E−07	30.14	4.0E−08	Additive
rs1329428	4.92	7.5E−04	4.0E−06	2.2E−06	23.34	1.4E−06	Recessive
rs1447295	4.08	9.5E−05	1.0E−04	1.1E−04	16.64	4.5E−05	Additive
rs698267	4.46	1.7E−05	2.1E−05	2.2E−05	19.70	9.0E−06	Additive
rs7837688	4.69	5.0E−06	7.0E−06	6.7E−06	22.36	2.3E−06	Additive
rs10510126	4.99	2.2E-16	2.0E−06	1.4E−06	22.95	1.7E−06	Recessive
rs12505080	4.15	8.2E−05	8.9E−05	8.5E−05	0.96	3.3E−01	Dominant
rs17157903	4.72	2.0E−06	9.0E−06	5.8E−06	11.68	6.3E−04	Heterosis
rs1219648	4.77	6.0E−06	1.0E−05	5.0E−06	23.45	1.3E−06	Additive
rs7696175	4.48	2.0E−05	3.3E−05	2.0E−05	0.30	5.8E−01	Heterosis
rs2420946	4.75	6.0E−06	8.0E−06	5.3E−06	23.20	1.5E−06	Additive
rs2820037	5.28	2.2E-16	2.2E-16	3.2E−07	16.12	5.9E−05	Heterosis
rs6997709	4.46	2.2E−05	3.3E−05	2.1E−05	20.00	7.7E−06	Additive
rs7961152	19.96	7.9E−06	1.8E−05	2.0E−05	16.12	5.9E−05	Additive
rs11110912	4.65	8.0E−06	1.1E−05	8.1E−06	19.48	1.0E−05	Recessive
rs1937506	4.43	2.4E−05	2.4E−05	2.4E−05	19.55	9.8E−06	Additive
rs2398162	4.91	1.0E−06	3.0E−06	2.4E−06	20.41	6.2E−06	Recessive

The underlying genetic models have been predicted using the MAX4 test procedure

A simulation study to investigate the significance of the MAX4 test in comparison with the allelic test was performed. A multinomial distribution was assumed for both cases and controls in violation of HWE, with model selection performed to investigate the underlying genetic models. Additional file 6: Table S5 shows a few selected most significant SNPs when the MAX4, using the asymptotic method, and the allelic tests were performed on the genetic models selected, at Bonferroni threshold of $10^{-5}$ with 5000 SNPs. The model selection predicted 2009 SNPs with the additive model, 2086 SNPs with the dominant model, 522 SNPs with the recessive model, and 383 SNPs with the heterotic model of the 5000 SNPs. There were 570 significant SNPs.

Table 3 shows the results of the MAX4 and allelic tests based on the 17 SNPs datasets (Additional file 2: Table S1). The performed model selection predicted the additive model with the highest proportion at 9 out of 17 SNPs. The proportions of the dominant and recessive models were at 1 and 4 of 17 SNPs, respectively. The heterotic model was selected at SNPs *rs*17157903, *rs*7696175, and *rs*2820037. Many SNPs returned significant results for the dominant, recessive, additive, and heterotic chi-square tests with more significance under the additive model compared with the other genetic models (Additional file 3: Figure S2). The *p*-values of the MAX4 test were estimated using the asymptotic method and it provided a similar approximation to the results of the parametric bootstrap and bivariate normal procedures as shown in Table 3. The *p*-values of some SNPs such as *rs*12505080 and *rs*7696175 reported a disparity between the MAX4 and the allelic tests.

**Table 4:** Frequency of heterotic models selected and the SNPs showing discordant results between the MAX4 and allelic test for Kenyan malaria datasets

Chr	SNPs	Heterosis	Cut-off	No.Discordant	No. Discordant/1000
X	693	436	0.0000722	17	39
Y	420	302	0.0001190	14	46
01	1696	1228	0.0000295	12	10
02	1159	781	0.0000431	8	10
03	1029	713	0.0000486	4	6
04	859	546	0.0000582	2	4
05	941	615	0.0000531	5	8
06	1528	1100	0.0000327	8	7
07	980	689	0.0000510	4	6
08	815	550	0.0000613	4	7
09	786	526	0.0000651	1	2
10	985	698	0.0000508	4	4
11	971	678	0.0000515	3	4
12	1060	7557	0.0000472	5	7
13	634	426	0.0000789	6	14
14	520	358	0.0000962	1	3
15	456	317	0.0001096	4	13
16	525	389	0.0000952	0	0
17	558	419	0.0000896	1	3
18	380	260	0.0001316	0	0
19	410	292	0.0001220	1	3
20	378	268	0.0001323	1	4
21	194	130	0.0002577	1	8
22	251	175	0.0001992	0	0

The cut-off is 0.05/number of SNPs per chromosome

### Real data

**Fig. 1:**
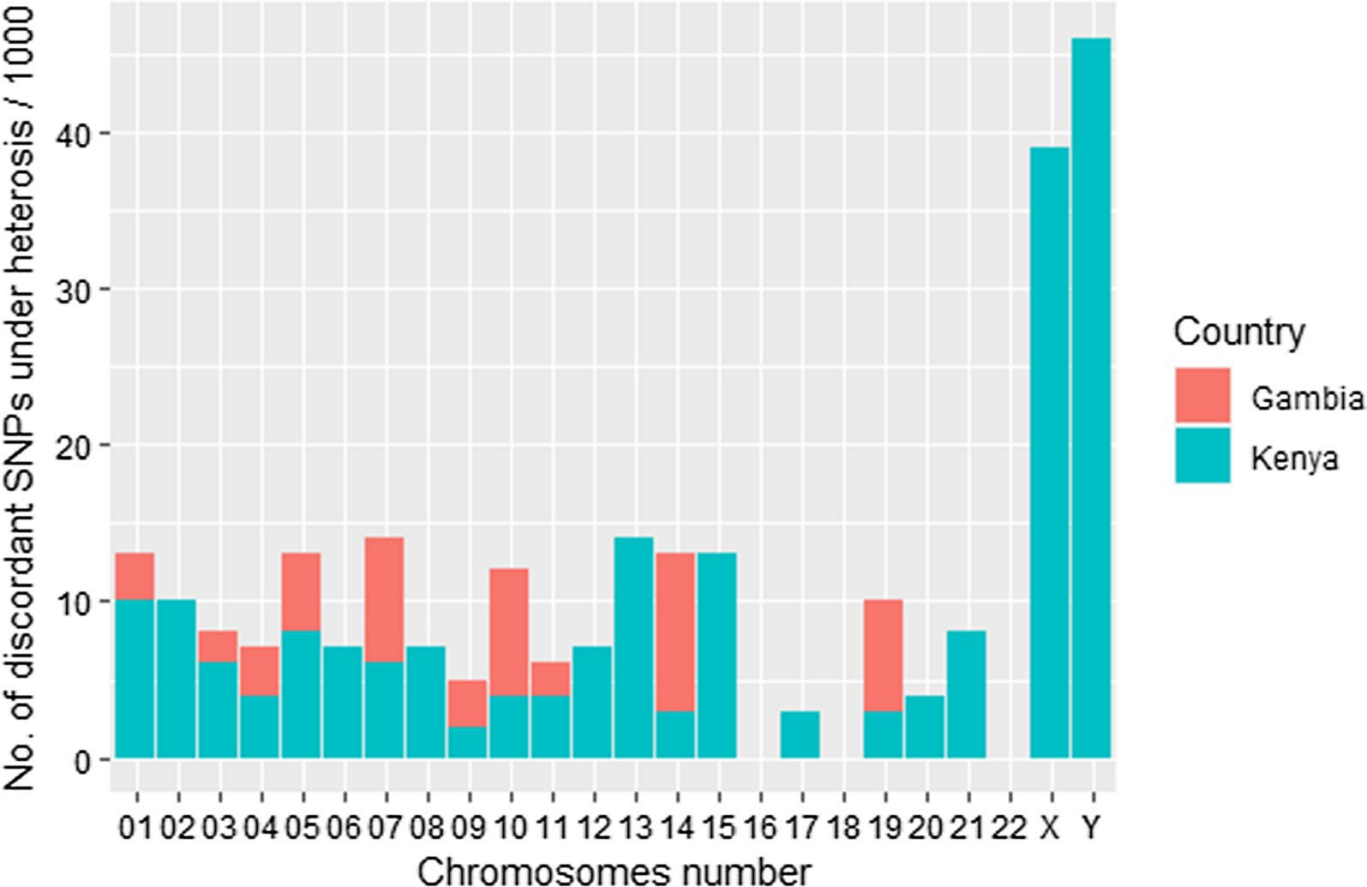
Results of disparity for the allelic and the MAX4 tests for the estimated heterotic models for Kenyan and Gambian malaria datasets

**Fig. 2:**
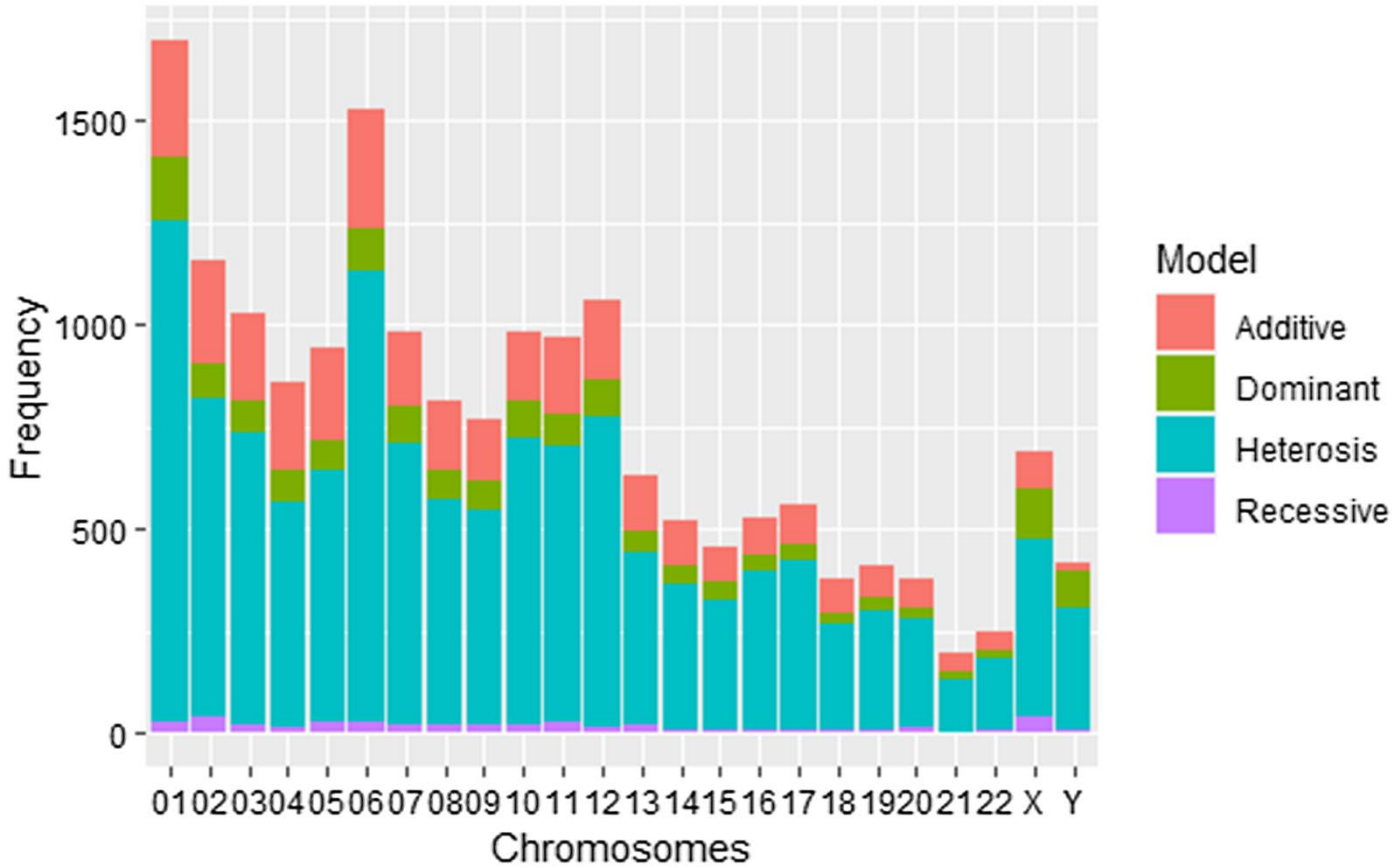
Frequency of different genetic modes of inheritances after performing MAX4 test for the model selection at allelic odds ratio greater than 1.5 for Kenyan malaria datasets

**Fig. 3:**
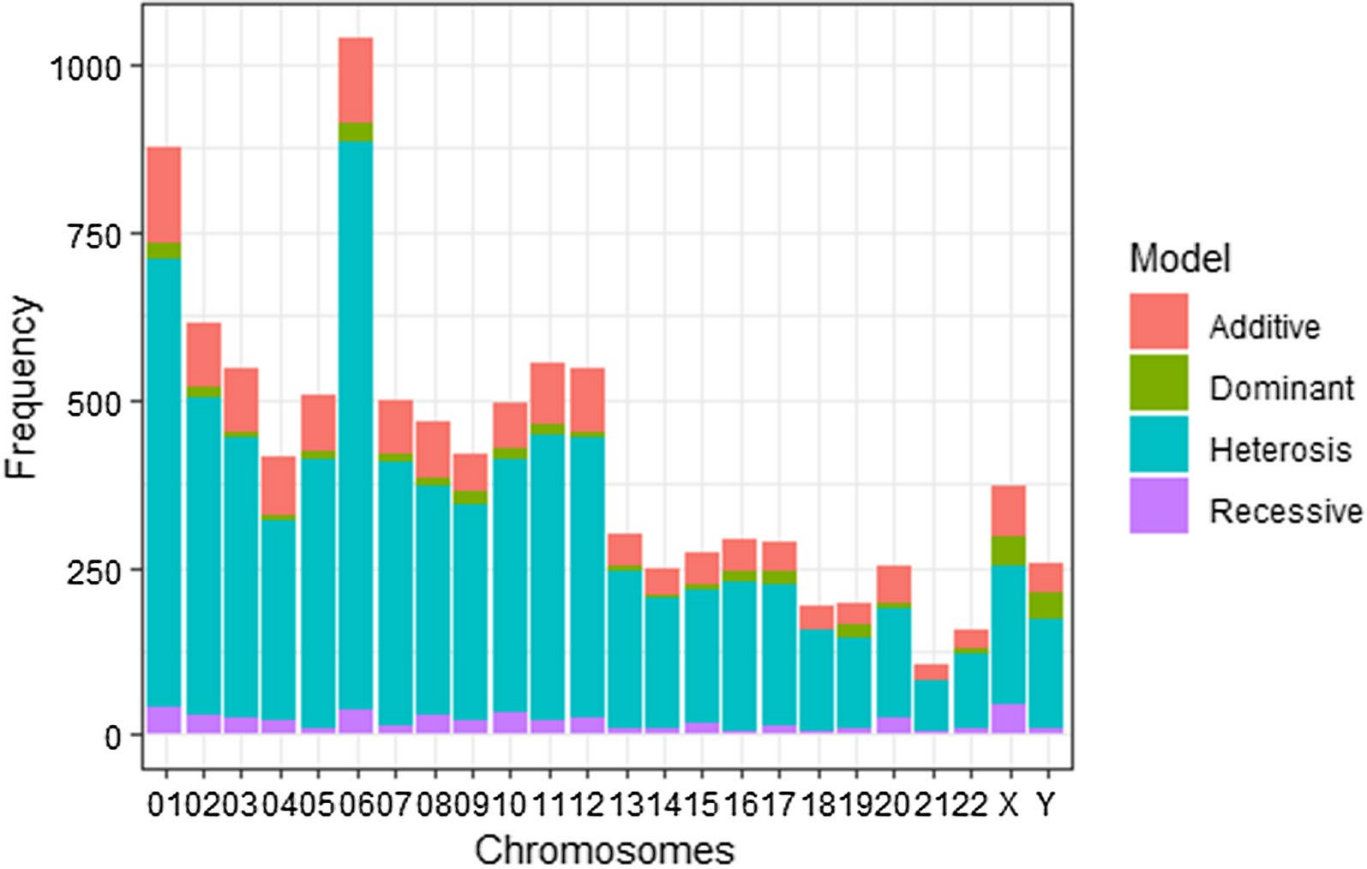
Frequency of different genetic modes of inheritances after performing MAX4 test for the model selection at allelic odds ratio greater than 1.5 for Gambian malaria datasets

In both the Kenyan and Gambian datasets, genome-wide significance is estimated using the conservative Bonferroni method, at an allelic odds ratio greater than or equal to 1.5. Tables 4 and 5 provide a summary of the frequency of heterotic models selected and disparities between the allelic test and the MAX4 test for Gambian and Kenyan populations, respectively. Discordance is when the standard MAX4 test results are not consistent with the allelic test results. For dominant, recessive, and additive models, there were no disparities between the two tests, i.e, both the MAX4 and allelic tests reported similar significant results (Additional file 4: Table S3 and Additional file 5: Table S4). Figure 1 shows heterotic frequencies and disparities between allelic and the MAX4 tests for Kenya and Gambia datasets. At allelic odds ratio greater than 1.5 ($95\%$ confidence interval), heterotic models reported the highest frequency. Figures 2 and 3 show the frequencies of the four genetic modes of inheritance selected using the MAX4 test procedure for Kenyan and Gambian datasets respectively. Manhattan plots and quantile-quantile (QQ) plots for selected chromosomes of Kenyan datasets are provided in additional information (Additional file 10: Fig. S1, Additional file 11: Fig. S2) and have been generated using the *qqman* package in R [26].

**Table 5:** Frequency of heterotic models selected and the SNPs showing discordant results between the MAX4 and allelic genome-wide Gambian malaria dataset

Chr	SNPs	Heterosis	Cut-off	No. Discordant	No. Discordant/1000
X	370	207	0.0001351	0	0
Y	256	161	0.0001953	0	0
01	876	667	0.0000571	2	3
02	615	473	0.0000813	0	0
03	547	419	0.0000914	1	2
04	417	298	0.0001199	1	3
05	506	399	0.0000988	2	5
06	1040	846	0.0000481	0	0
07	497	391	0.0001006	3	8
08	467	342	0.0001071	0	0
09	420	322	0.0001190	1	3
10	495	379	0.0001010	3	8
11	553	423	0.0000904	1	2
12	547	416	0.0000914	0	0
13	298	233	0.0001678	0	0
14	250	195	0.0002000	2	10
15	271	200	0.0001845	0	0
16	292	223	0.0001712	0	0
17	289	213	0.0001730	0	0
18	192	148	0.0002604	0	0
19	196	136	0.0002551	1	7
20	251	161	0.0001992	0	0
21	103	74	0.0004854	0	0
22	155	109	0.0003226	0	0

The cut-off is 0.05/number of SNPs per chromosome. Many chromosomes reported no disparity between the two tests

## Discussion

The study assessed the performance of the MAX4 and the allelic tests in malaria studies. The test, known as the MAX, has been previously used in genetic association testing ( [9, 12, 27]). The test allowed for model selection as well as testing of statistical significance. The MAX4 test was the standard test procedure since deviations from its conclusions were deemed false negative by the allelic test. The test is a robust test procedure that allows for genetic and other covariates in the analysis since it incorporates the generalized linear model and has good power and model selection properties [9].

One of the significant findings from the GWAS analyses was the uneven distribution of the disparities in the association test results between the MAX4 test and the allelic test across the chromosomes (Tables 4,5 and Fig. 1). It turned out that the highest disparities occurred in chromosomes X and Y in the Kenyan dataset. Disparities were also observed in chromosomes 1, 2, 13, and 15 (Kenyan dataset) and chromosome 14 (Gambian dataset). The 17 SNPs dataset in Table 3 also reported disparities for SNPs rs12505080 and rs7696175.

Figures 2 and 3 show the highest frequencies at chromosomes 1 and 6 in both Kenyan and Gambian datasets. The two chromosomes also have the most heterotic pattern of inheritance. Chromosomes 1 and 6 have previously been investigated and proven to be protective against severe malaria [28–30].

All SNPs were tested for compliance with the HWE before genetic association testing. It was noted that the prevalence of heterotic associations was higher in the Kenyan dataset than the Gambian dataset, further highlighting the genetic diversity between the two populations from the Eastern and Western regions of Africa, respectively. Recent GWAS have implicated chromosome 6 with the SNPs associated with drug-resistant to severe malaria [31]. The recommendation of further studies to be conducted on the chromosomes above to assess their association with malaria protection is required, given the presence of significant heterotic effects in these chromosomes. These results support the findings of simulation studies by Omolo et al. [3], which found that the allelic tests lose power in the presence of heterosis, resulting in false-negative results.

Existing research in single-SNP and genome-wide studies tend to overlook overdominance and underdominance, even though the circumstances reduce the power of allelic tests [3]. The research findings are consistent with simulation study results, which recommended performing the allelic test with care for single SNPs in the presence of heterosis due to power loss.

## Conclusion

Based on simulation studies conducted by Omolo et al. [3], who cautioned against overlooking heterotic conditions when performing allelic tests because it resulted in power loss in the presence of the condition, the findings hold in both single SNP analysis and genome-wide association studies. Statistical methods in previous studies examined popular genetic models but ignored heterosis, even though the power of allelic tests reduced in the presence of heterosis.

## Supplementary Information

**Additional file 1: Text S1.** Distribution of Z under overdominance.

**Additional file 2: Table S1.** Genotype distributions of the 17 SNPs selected from GWAS; Age-related macular degeneration(AMD), Prostate Cancer (PC), Breast cancer(BC) and Hypertension(HP).

**Addition file 3: Table S2.** Chi-square tests and the MAX test results of the 17 SNPs selected from the GWAS; Age-related macular degeneration (AMD), Prostate Cancer (PC), Breast cancer (BC) and Hypertension (HP). The chi-square tests were performed under dominant(DOM), recessive (REC), additive (ADD), and heterotic (HET) models. The genotype test was also performed and the results shown.

**Additional file 4: Table S3.** The MAX4 and the ABT tests were performed on the Gambian datasets for the additive, recessive, and dominant models. Results show there was no disparity between the two tests.

**Additional file 5: Table S4.** The MAX4 and the ABT tests were performed on the Kenyan datasets for the additive, recessive, and dominant models. Results show there was no disparity between the two tests.

**Additional file 6: Table S5.** Simulation results of some most significant SNPs selected.

**Additional file 7: Text S2.** R codes for MAX4 the and Allelic test for Kenyan datasets.

**Additional file 8: Text S3.** Manhattan and quantile-quantile plots for selected chromosomes for Kenyan datasets.

**Additional file 9: Text S4.** Simulation codes.

**Additional file 10: Figure S1.** Manhattan plots of association findings for additive, dominant, recessive, and overdominant models for selected chromosomes. The MAX4 test performs model selection using the *P*-value approach for the Kenyan datasets.

**Additional file 11: Figure S2.** Quantile-quantile plots of association findings for additive, dominant, recessive, and overdominant models for selected chromosomes. The MAX test performs model selection using the *P*-value approach for the Kenyan datasets.

## Data Availability

The data is available upon request from the MalariaGEN network. *R* software was used.

## References

[1] Hedrick PW (2011). Population genetics of malaria resistance in humans. Heredity..

[2] Comings D (1999). Molecular heterosis as the explanation for the controversy about the effect of the DRD2 gene on dopamine D2 receptor density. Mol Psychiatry..

[3] Omolo B, Zhang H, Karmaus W (2013). Cautions of using allele-based tests under heterosis. Int J Stat Med Res..

[4] Gail MH, Pee D, Benichou J, Carroll R (1999). Designing studies to estimate the penetrance of an identified autosomal dominant mutation: cohort, case-control, and genotyped-proband designs. Genet Epidemiol..

[5] Amos CI (2007). Successful design and conduct of genome-wide association studies. Hum Mol Genet..

[6] Uffelmann E, Huang QQ, Munung NS, de Vries J, Okada Y, Martin AR (2021). Genome-wide association studies. Nat Rev Methods Primers..

[7] Zheng G, Joo J, Yang Y (2009). Pearson’s test, trend test, and MAX are all trend tests with different types of scores. Ann Hum Genet..

[8] Sasieni PD. From genotypes to genes: doubling the sample size. Biometrics. 1997;p. 1253–1261. Available from: http://www.jstor.org/stable/2533494. 9423247

[9] Loley C, König IR, Hothorn L, Ziegler A (2013). A unifying framework for robust association testing, estimation, and genetic model selection using the generalized linear model. Eur J Hum Genet..

[10] González JR, Carrasco JL, Dudbridge F, Armengol L, Estivill X, Moreno V (2008). Maximizing association statistics over genetic models. Genet Epidemiol..

[11] Zang Y, Fung WK, Zheng G (2010). Simple algorithms to calculate the asymptotic null distributions of robust tests in case-control genetic association studies in R. J Stat Softw..

[12] Hothorn LA, Hothorn T (2009). Order-restricted scores test for the evaluation of population-based case-control studies when the genetic model is unknown. Biom J..

[13] Zintzaras E, Santos M (2011). Estimating the mode of inheritance in genetic association studies of qualitative traits based on the degree of dominance index. BMC Med Res Methodol..

[14] Strasser H, Weber C. On the asymptotic theory of permutation statistics. 1999.

[15] Dimou NL, Tsirigos KD, Elofsson A, Bagos PG (2017). GWAR: robust analysis and meta-analysis of genome-wide association studies. Bioinformatics..

[16] Li G, Zhu H (2013). Genetic Studies: The Linear Mixed Models in Genome-wide Association Studies. Open Bioinform J..

[17] Joo J, Kwak M, Chen Z, Zheng G (2010). Efficiency robust statistics for genetic linkage and association studies under genetic model uncertainty. Stat Med..

[18] Klein RJ, Zeiss C, Chew EY, Tsai JY, Sackler RS, Haynes C (2005). Complement factor H polymorphism in age-related macular degeneration. Science..

[19] Hunter DJ, Kraft P, Jacobs KB, Cox DG, Yeager M, Hankinson SE (2007). A genome-wide association study identifies alleles in FGFR2 associated with risk of sporadic postmenopausal breast cancer. Nat Genet..

[20] Yeager M, Orr N, Hayes RB, Jacobs KB, Kraft P, Wacholder S (2007). Genome-wide association study of prostate cancer identifies a second risk locus at 8q24. Nat Genet..

[21] Consortium WTCC (2007). Genome-wide association study of 14,000 cases of seven common diseases and 3000 shared controls. Nature..

[22] Band G, Le QS, Jostins L, Pirinen M, Kivinen K, Jallow M (2013). Imputation-based meta-analysis of severe malaria in three African populations. PLoS Genet..

[23] Team R, et al. RStudio: integrated development for R. RStudio, Inc, Boston, MA URL http://wwwrstudiocom. 2015;42:84.

[24] Freidlin B, Zheng G, Li Z, Gastwirth JL (2009). Trend tests for case-control studies of genetic markers: power, sample size and robustness. Hum Hered..

[25] Sloane D, Morgan SP (1996). An introduction to categorical data analysis. Annu Rev Sociol..

[26] Turner SD. qqman: an R package for visualizing GWAS results using QQ and manhattan plots. Biorxiv. 2014;p. 005165.

[27] Zheng G, Meyer M, Li W, Yang Y (2008). Comparison of two-phase analyses for case-control genetic association studies. Stat Med..

[28] Brisebarre A, Kumulungui B, Sawadogo S, Atkinson A, Garnier S, Fumoux F (2014). A genome scan for Plasmodium falciparum malaria identifies quantitative trait loci on chromosomes 5q31, 6p213, 17p12, and 19p13. Malar J..

[29] Flori L, Sawadogo S, Esnault C, Delahaye NF, Fumoux F, Rihet P (2003). Linkage of mild malaria to the major histocompatibility complex in families living in Burkina Faso. Hum Mol Genet..

[30] Timmann C, Evans JA, König IR, Kleensang A, Rüschendorf F, Lenzen J (2007). Genome-wide linkage analysis of malaria infection intensity and mild disease. PLoS Genet..

[31] Network MGE (2019). Insights into malaria susceptibility using genome-wide data on 17,000 individuals from Africa, Asia and Oceania. Nat Commun..

