# Supplementary material for: Genome-wide association testing in malaria studies in the presence of overdominance
Source: Malar J. 2023 Apr 10;22:119. doi: 10.1186/s12936-023-04533-2 (PMC10084622; doi:10.1186/s12936-023-04533-2)
Supplement: Supplementary file 1 — Additional file 1: Text S1. Distribution of Z under overdominance. [file 12936_2023_4533_MOESM1_ESM.docx]

**Genome-wide association testing in malaria studies in the presence of overdominance.**

Additional File1:Text S1 Distribution of Z under overdominance

$$U=\sum_{i=0}^{2} x{}_{i}(Sr_{i}-Rs_{i})$$

Given that $S=N-R$

$$Var(U)=Var(\sum_{i=0}^{2} x{}_{i}(Sr_{i}-Rs_{i}))$$

$\begin{matrix} =S^{2}[\sum_{i=0}^{2} x_{i}^{2}Var(r_{i})+ 2\sum_{i=0}^{2} \sum_{j=i+1}^{2} x_{i}x_{j}\mathrm{cov}(r_{i}r_{j}) ]+ \\ R^{2}[\sum_{i=0}^{2} x_{i}^{2}Var(s_{i})+ 2\sum_{i=0}^{2} \sum_{j=i+1}^{2} x_{i}x_{j}\mathrm{cov}(s_{i}s_{j}) ] \end{matrix}$

Under the null hypothesis and overdominance model,

 [1]

For large N $\frac{U}{Var(U)}$ ~$N(0,1)$ [2]

The same procedure can be used to derive the asymptotic variance under the dominant, recessive, and additive models.
