# Supplementary material for: Genome-wide association testing in malaria studies in the presence of overdominance
Source: Malar J. 2023 Apr 10;22:119. doi: 10.1186/s12936-023-04533-2 (PMC10084622; doi:10.1186/s12936-023-04533-2)
Supplement: Supplementary file 2 — Additional file 2: Table S1. Genotype distributions of the 17 SNPs selected from GWAS; Age-related macular degeneration(AMD), Prostate Cancer (PC), Breast cancer(BC) and Hypertension(HP). [file 12936_2023_4533_MOESM2_ESM.docx]

Additional File 2: Table S1 Genotype distributions of the 17 SNPs selected from the GWAS, Age-related macular degeneration (AMD) [1], Prostate Cancer (PC) [2], Breast cancer (BC) [3] and Hypertension (HP) [4]

Cases and Controls

SNP ID Casesdd CasesdD CasesDD Controldd ControldD ControlDD

*Rs380390*(AMD) 50 35 11 6 25 19

*Rs1329428*(AMD) 2 24 68 5 29 14

*Rs1447295*(PC) 25 283 864 10 218 929

*Rs698267*(PC) 223 598 351 301 579 277

*Rs7837688*(PC) 27 283 861 11 206 939

*Rs10510126*(PC) 10 180 955 14 272 854

*Rs12505080*(BC) 50 477 608 99 408 628 *Rs17157903*(BC) 18 316 777 26 220 862 *Rs1219648*(BC) 250 543 352 170 538 433

*Rs7696175*(BC) 187 605 353 249 496 396

*Rs2420946*(BC) 242 546 357 165 537 440

*Rs2820037*(HP) 40 587 1,325 72 684 2,180

*Rs6997709*(HP) 118 716 1,116 237 1,201 1,500

*Rs7961152*(HP) 416 963 570 492 1,448 992

*Rs11110912*(HP) 67 647 1,237 83 804 2,049

*Rs1937506*(HP) 113 742 1,097 244 1,205 1,484

*Rs2398162*(HP) 111 624 1,205 194 1,121 1,608
