# Supplementary material for: Genome-wide association testing in malaria studies in the presence of overdominance
Source: Malar J. 2023 Apr 10;22:119. doi: 10.1186/s12936-023-04533-2 (PMC10084622; doi:10.1186/s12936-023-04533-2)
Supplement: Supplementary file 3 — Addition file 3: Table S2. Chi-square tests and the MAX test results of the 17 SNPs selected from the GWAS; Age-related macular degeneration (AMD), Prostate Cancer (PC), Breast cancer (BC) and Hypertension (HP). The chi-square tests were performed under dominant(DOM), recessive (REC), additive (ADD), and heterotic (HET) models. The genotype test was also performed and the results shown. [file 12936_2023_4533_MOESM3_ESM.docx]

Additional File 3: Table S2 Chi-square tests and the MAX test results of the 17 SNPs selected from the GWAS, Age-related macular degeneration (AMD) [1], Prostate Cancer (PC) [2], Breast cancer (BC) [3] and Hypertension (HP) [4]. The chi-square test was performed under dominant(DOM), recessive(REC), additive(ADD) and heterotic (HET) models. The genotype test was also performed.

| SNP ID | MODEL |  | MAXpval | DOM_pval | REC_pval | ADD_pval | HET_pval | Genotype_pv |
| --- | --- | --- | --- | --- | --- | --- | --- | --- |
| *rs380390* | Additive |  | 8.56E-07 | 6.17E-06 | 0.000418 | 8.56E-07 | 0.216247 | 1.80E-06 |
| *rs1329428* | Recessive |  | 2.21E-06 | 0.066268 | 2.21E-06 | 2.30E-06 | 0.000121 | 3.60E-06 |
| *rs1447295* | Additive |  | 0.000109 | 0.026024 | 0.000391 | 0.000109 | 0.004222 | 1.90E-04 |
| *rs6983267* | Additive |  | 2.16E-05 | 0.000143 | 0.002698 | 2.16E-05 | 0.865484 | 3.50E-05 |
| *rs7837688* | Additive |  | 6.66E-06 | 0.022018 | 2.24E-05 | 6.66E-06 | 0.00041 | 1.60E-05 |
| *rs10510126* | Recessive |  | 1.41E-06 | 0.650767 | 1.41E-06 | 3.37E-06 | 2.54E-06 | 3.70E-06 |
| *rs12505080* | Dominant |  | 8.46E-05 | 8.46E-05 | 0.639791 | 0.544965 | 0.007032 | 1.80E-05 |
| *rs17157903* | Heterosis |  | 5.76E-06 | 0.397101 | 6.17E-05 | 0.001492 | 5.76E-06 | 9.90E-06 |
| *rs1219648* | Additive |  | 4.99E-06 | 4.97E-05 | 0.000726 | 4.99E-06 | 0.988946 | 7.50E-06 |
| *rs7696175* | Heterosis |  | 2.01E-05 | 0.002069 | 0.100329 | 0.824674 | 2.01E-05 | 1.60E-05 |
| *rs2420946* | Additive |  | 5.34E-06 | 7.72E-05 | 0.000577 | 5.34E-06 | 0.936387 | 8.80E-06 |
| *rs2820037* | Heterosis |  | 3.17E-07 | 0.588908 | 3.23E-06 | 0.000141 | 3.17E-07 | 7.70E-07 |
| *rs6997709* | Additive |  | 2.07E-05 | 0.017889 | 5.83E-05 | 2.07E-05 | 0.008284 | 4.40E-05 |
| *rs7961152* | Additive |  | 2.01E-05 | 0.000158 | 0.00191 | 2.01E-05 | 0.999826 | 3.00E-05 |
| *rs11110912* | Recessive |  | 8.15E-06 | 0.409801 | 8.15E-06 | 2.32E-05 | 3.75E-05 | 1.90E-05 |
| *rs1937506* | Additive |  | 2.43E-05 | 0.002133 | 0.00031 | 2.43E-05 | 0.067814 | 4.50E-05 |
| *rs2398162* | Recessive |  | 2.42E-06 | 0.359548 | 2.42E-06 | 2.05E-05 | 2.77E-05 | 5.70E-06 |
|  |  |  |  |  |  |  |  |  |
