# Supplementary material for: Genome-wide association testing in malaria studies in the presence of overdominance
Source: Malar J. 2023 Apr 10;22:119. doi: 10.1186/s12936-023-04533-2 (PMC10084622; doi:10.1186/s12936-023-04533-2)
Supplement: Supplementary file 4 — Additional file 4: Table S3. The MAX4 and the ABT tests were performed on the Gambian datasets for the additive, recessive, and dominant models. Results show there was no disparity between the two tests. [file 12936_2023_4533_MOESM4_ESM.docx]

| *Additional File 4: Table S3 The MAX4 and the ABT tests were performed on the Gambian datasets for the additive, recessive, and dominant models. Results show there was no disparity between the two tests*   \| CHR \| Total \| Cutoff \| Freq_Dom \| Disparity \| Prop \| Freq_Rec \| Disparity \| Prop \| Freq_Add \| Disparity \| Prop \| \| --- \| --- \| --- \| --- \| --- \| --- \| --- \| --- \| --- \| --- \| --- \| --- \| \| X \| 370 \| 0.0001351 \| 44 \| 0 \| 0 \| 45 \| 0 \| 0 \| 74 \| 0 \| 0 \| \| Y \| 256 \| 0.0001953 \| 41 \| 0 \| 0 \| 10 \| 0 \| 0 \| 44 \| 0 \| 0 \| \| 01 \| 876 \| 0.0000571 \| 27 \| 0 \| 0 \| 41 \| 0 \| 0 \| 141 \| 0 \| 0 \| \| 02 \| 615 \| 0.0000813 \| 16 \| 0 \| 0 \| 28 \| 0 \| 0 \| 98 \| 0 \| 0 \| \| 03 \| 547 \| 0.0000914 \| 8 \| 0 \| 0 \| 24 \| 0 \| 0 \| 96 \| 0 \| 0 \| \| 04 \| 417 \| 0.0001199 \| 8 \| 0 \| 0 \| 22 \| 0 \| 0 \| 89 \| 0 \| 0 \| \| 05 \| 506 \| 0.0000988 \| 14 \| 0 \| 0 \| 11 \| 0 \| 0 \| 82 \| 0 \| 0 \| \| 06 \| 1040 \| 0.0000481 \| 27 \| 0 \| 0 \| 39 \| 0 \| 0 \| 128 \| 0 \| 0 \| \| 07 \| 497 \| 0.0001006 \| 14 \| 0 \| 0 \| 15 \| 0 \| 0 \| 77 \| 0 \| 0 \| \| 08 \| 467 \| 0.0001071 \| 10 \| 0 \| 0 \| 30 \| 0 \| 0 \| 85 \| 0 \| 0 \| \| 09 \| 420 \| 0.000119 \| 19 \| 0 \| 0 \| 21 \| 0 \| 0 \| 58 \| 0 \| 0 \| \| 10 \| 495 \| 0.000101 \| 15 \| 0 \| 0 \| 32 \| 0 \| 0 \| 69 \| 0 \| 0 \| \| 11 \| 553 \| 0.0000904 \| 19 \| 0 \| 0 \| 22 \| 0 \| 0 \| 89 \| 0 \| 0 \| \| 12 \| 547 \| 0.0000914 \| 10 \| 0 \| 0 \| 26 \| 0 \| 0 \| 95 \| 0 \| 0 \| \| 13 \| 298 \| 0.0001678 \| 10 \| 0 \| 0 \| 10 \| 0 \| 0 \| 45 \| 0 \| 0 \| \| 14 \| 250 \| 0.0002 \| 5 \| 0 \| 0 \| 8 \| 0 \| 0 \| 42 \| 0 \| 0 \| \| 15 \| 271 \| 0.0001845 \| 7 \| 0 \| 0 \| 16 \| 0 \| 0 \| 48 \| 0 \| 0 \| \| 16 \| 292 \| 0.0001712 \| 15 \| 0 \| 0 \| 7 \| 0 \| 0 \| 47 \| 0 \| 0 \| \| 17 \| 289 \| 0.000173 \| 17 \| 0 \| 0 \| 13 \| 0 \| 0 \| 46 \| 0 \| 0 \| \| 18 \| 192 \| 0.0002604 \| 2 \| 0 \| 0 \| 7 \| 0 \| 0 \| 35 \| 0 \| 0 \| \| 19 \| 196 \| 0.0002551 \| 19 \| 0 \| 0 \| 10 \| 0 \| 0 \| 31 \| 0 \| 0 \| \| 20 \| 251 \| 0.0001992 \| 9 \| 0 \| 0 \| 26 \| 0 \| 0 \| 55 \| 0 \| 0 \| \| 21 \| 103 \| 0.0004854 \| 2 \| 0 \| 0 \| 7 \| 0 \| 0 \| 20 \| 0 \| 0 \| \| 22 \| 155 \| 0.0003226 \| 9 \| 0 \| 0 \| 11 \| 0 \| 0 \| 26 \| 0 \| 0 \| |  |  |  |  |  |  |  |  |  |  |  |  |  |  |  |
| --- | --- | --- | --- | --- | --- | --- | --- | --- | --- | --- | --- | --- | --- | --- | --- | --- | --- | --- | --- | --- | --- | --- | --- | --- | --- | --- | --- | --- | --- | --- | --- | --- | --- | --- | --- | --- | --- | --- | --- | --- | --- | --- | --- | --- | --- | --- | --- | --- | --- | --- | --- | --- | --- | --- | --- | --- | --- | --- | --- | --- | --- | --- | --- | --- | --- | --- | --- | --- | --- | --- | --- | --- | --- | --- | --- | --- | --- | --- | --- | --- | --- | --- | --- | --- | --- | --- | --- | --- | --- | --- | --- | --- | --- | --- | --- | --- | --- | --- | --- | --- | --- | --- | --- | --- | --- | --- | --- | --- | --- | --- | --- | --- | --- | --- | --- | --- | --- | --- | --- | --- | --- | --- | --- | --- | --- | --- | --- | --- | --- | --- | --- | --- | --- | --- | --- | --- | --- | --- | --- | --- | --- | --- | --- | --- | --- | --- | --- | --- | --- | --- | --- | --- | --- | --- | --- | --- | --- | --- | --- | --- | --- | --- | --- | --- | --- | --- | --- | --- | --- | --- | --- | --- | --- | --- | --- | --- | --- | --- | --- | --- | --- | --- | --- | --- | --- | --- | --- | --- | --- | --- | --- | --- | --- | --- | --- | --- | --- | --- | --- | --- | --- | --- | --- | --- | --- | --- | --- | --- | --- | --- | --- | --- | --- | --- | --- | --- | --- | --- | --- | --- | --- | --- | --- | --- | --- | --- | --- | --- | --- | --- | --- | --- | --- | --- | --- | --- | --- | --- | --- | --- | --- | --- | --- | --- | --- | --- | --- | --- | --- | --- | --- | --- | --- | --- | --- | --- | --- | --- | --- | --- | --- | --- | --- | --- | --- | --- | --- | --- | --- | --- | --- | --- | --- | --- | --- | --- | --- | --- | --- | --- | --- | --- | --- | --- | --- | --- | --- | --- | --- | --- | --- | --- | --- | --- | --- | --- | --- | --- | --- | --- | --- | --- | --- | --- | --- | --- | --- | --- | --- | --- | --- | --- | --- | --- | --- |
|  |  |  |  |  |  |  |  |  |  |  |  |  |  |  |  |
|  |  |  |  |  |  |  |  |  |  |  |  |  |  |  |  |
|  |  |  |  |  |  |  |  |  |  |  |  |  |  |  |  |
|  |  |  |  |  |  |  |  |  |  |  |  |  |  |  |  |
|  |  |  |  |  |  |  |  |  |  |  |  |  |  |  |  |
|  |  |  |  |  |  |  |  |  |  |  |  |  |  |  |  |
|  |  |  |  |  |  |  |  |  |  |  |  |  |  |  |  |
|  |  |  |  |  |  |  |  |  |  |  |  |  |  |  |  |
|  |  |  |  |  |  |  |  |  |  |  |  |  |  |  |  |
|  |  |  |  |  |  |  |  |  |  |  |  |  |  |  |  |
