# Supplementary material for: Genome-wide association testing in malaria studies in the presence of overdominance
Source: Malar J. 2023 Apr 10;22:119. doi: 10.1186/s12936-023-04533-2 (PMC10084622; doi:10.1186/s12936-023-04533-2)
Supplement: Supplementary file 6 — Additional file 6: Table S5. Simulation results of some most significant SNPs selected. [file 12936_2023_4533_MOESM6_ESM.docx]

*Additional File 6: Table S5 Simulation results of some most significant SNPs selected.*

| AA | Aa | aa | AA | Aa | aa | Mode; | Max_pval | Allelic_pval |
| --- | --- | --- | --- | --- | --- | --- | --- | --- |
| 348 | 496 | 239 | 417 | 883 | 408 | Dominant | 2.28E-05 | 0.00051 |
| 685 | 1021 | 519 | 649 | 1286 | 658 | Dominant | 2.30E-05 | 0.000132 |
| 220 | 292 | 145 | 728 | 1483 | 702 | Dominant | 2.30E-05 | 0.000567 |
| 152 | 213 | 98 | 66 | 192 | 92 | Dominant | 2.30E-05 | 0.000137 |
| 416 | 674 | 308 | 435 | 937 | 500 | Additive | 2.35E-05 | 7.45E-06 |
| 423 | 665 | 303 | 207 | 485 | 246 | Dominant | 2.39E-05 | 1.85E-05 |
| 434 | 758 | 304 | 583 | 1260 | 620 | Additive | 2.42E-05 | 1.09E-05 |
| 865 | 1354 | 620 | 471 | 973 | 473 | Dominant | 2.44E-05 | 2.87E-05 |
| 899 | 1369 | 598 | 379 | 748 | 373 | Additive | 2.46E-05 | 6.98E-06 |
| 296 | 458 | 188 | 783 | 1586 | 789 | Additive | 2.49E-05 | 8.95E-06 |
| 400 | 679 | 257 | 591 | 1175 | 591 | Additive | 2.49E-05 | 9.71E-06 |
| 845 | 1306 | 576 | 257 | 530 | 274 | Additive | 2.51E-05 | 7.07E-06 |
| 387 | 567 | 248 | 145 | 336 | 166 | Dominant | 2.52E-05 | 1.68E-05 |
| 307 | 454 | 203 | 809 | 1644 | 823 | Dominant | 2.53E-05 | 1.49E-05 |
| 522 | 859 | 386 | 697 | 1420 | 750 | Additive | 2.57E-05 | 8.04E-06 |
| 1012 | 1745 | 718 | 228 | 431 | 250 | Recessive | 2.59E-05 | 3.50E-05 |
| 116 | 139 | 101 | 436 | 939 | 435 | Heterosis | 2.59E-05 | 0.310435 |
| 457 | 724 | 334 | 708 | 1491 | 748 | Dominant | 2.65E-05 | 2.24E-05 |
| 383 | 613 | 271 | 266 | 575 | 312 | Additive | 2.66E-05 | 8.22E-06 |
| 252 | 359 | 161 | 489 | 1015 | 503 | Dominant | 2.68E-05 | 3.04E-05 |
| 600 | 867 | 424 | 504 | 1022 | 464 | Dominant | 2.68E-05 | 0.001289 |
| 211 | 315 | 142 | 583 | 1284 | 640 | Dominant | 2.70E-05 | 4.25E-05 |
