# Supplementary material for: Genome-wide association testing in malaria studies in the presence of overdominance
Source: Malar J. 2023 Apr 10;22:119. doi: 10.1186/s12936-023-04533-2 (PMC10084622; doi:10.1186/s12936-023-04533-2)
Supplement: Supplementary file 7 — Additional file 7: Text S2. R codes for MAX4 the and Allelic test for Kenyan datasets. [file 12936_2023_4533_MOESM7_ESM.docx]

Additional File 7: Text S2: R codes for MAX TEST AND ALLELIC TESTS ( MAX4 EXTENDED TO HETEROTIC GENETIC MODEL) for Kenyan datasets

Both Kenyan and Gambian datasets from malariaGEN were pre-processed using SNP test V 2.4.1 then organized into 2 by 3 case-control tables before applying the MAX and the allelic tests using Rassoc package [5] [6].

setwd("D:/tests - 904")

data=read.csv("ch11New.csv")

#install.packages("readxl")

#library("readxl")

#data <- read_excel("ch1filtered.xlsx")

names(data)[5]='cases_AA'

names(data)[6]='cases_AB'

names(data)[7]='cases_BB'

names(data)[8]='controls_AA'

data

p=apply(data,2,sum)/sum(data)

p0=p[1]

p1=p[2]

p2=p[3]

rho005=p2*(p1+2*p0)/(sqrt(p2*(1-p2))*sqrt((p1+2*p2)*p0+(p1+2*p0)*p2))

rho105=p0*(p1+2*p2)/(sqrt(p0*(1-p0))*sqrt((p1+2*p2)*p0+(p1+2*p0)*p2))

rho01=sqrt((p0*p2)/((1-p0)*(1-p2)))

w0=(rho005-rho01*rho105)/(1-rho01^2)

w1=(rho105-rho01*rho005)/(1-rho01^2)

r=sum(data[1,])

s=sum(data[2,])

## Define CATT. ##

CATT=function(tab,score){

nr=apply(tab,2,sum)

n=sum(nr)

Rbar=sum(nr*score)/n

s2=sum(nr*(score-Rbar)^2)

phi=sum(tab[1,])/n

catt_v=sum(tab[1,]*(score-Rbar))/sqrt(phi*(1-phi)*s2)

## Report the statistic of CATT. ##

return(catt_v)

}

## Report the statistic of MAX3. ##

dom=abs(CATT(data,c(0,1,1)))

rec=abs(CATT(data,c(0,0,1)))

add=abs(CATT(data,c(0,0.5,1)))

het=abs(CATT(data,c(0,1,0)))

maxr=max(dom,rec,add,het)

if((method!="boot")&&(method!="bvn")&&(method!="asy"))

stop("method must be boot, bvn or asy.")

## Use "boot" to calculate the p-value of MAX3. ##

if(method=="boot"){

CATTN=function(data,score){

tab=matrix(data,nrow=2,byrow=TRUE)

nr=apply(tab,2,sum)

n=sum(nr)

Rbar=sum(nr*score)/n

s2=sum(nr*(score-Rbar)^2)

phi=sum(tab[1,])/n

catt_v=sum(tab[1,]*(score-Rbar))/sqrt(phi*(1-phi)*s2)

return(catt_v)

}

ca=rmultinom(m,r,p)

co=rmultinom(m,s,p)

caco=rbind(ca,co)

caco0=apply(caco,2,CATTN,score=c(0,0,1))

caco05=apply(caco,2,CATTN,score=c(0,0.5,1))

caco1=apply(caco,2,CATTN,score=c(0,1,1))

cacoH=apply(caco,2,CATTN,score=c(0,1,0))

cacos=rbind(abs(caco0),abs(caco05),abs(caco1), cacoH)

max1=apply(cacos,2,max)

## Report empirical p-value using "boot" method. ##

pv=length(max1[max1>=maxr])/m

pvdom=length(max1[max1>=dom])/m

pvrec=length(max1[max1>=rec])/m

pvadd=length(max1[max1>=add])/m

pvhet=length(max1[max1>=het])/m

md="The MAX3 test using the boot method"

}

## Use "bvn" to caculate the p-value of MAX3. ##

if(method=="bvn"){

BV05=function(x,a1,a2){

return(a1*x[1]+a2*x[2])

}

bv01=rmvnorm(m,mean=c(0,0),sigma=matrix(c(1,rho01,rho01,1),nrow=2))

bv05=apply(bv01,1,BV05,a1=w0,a2=w1)

bv=cbind(abs(bv01),abs(bv05))

max2=apply(bv,1,max)

## Report empirical p-value using "bvn" method. ##

pv=length(max2[max2>=maxr])/m

pvdom=length(max2[max2>=dom])/m

pvrec=length(max2[max2>=rec])/m

pvadd=length(max2[max2>=add])/m

pvhet=length(max2[max2>=het])/m

md="The MAX3 test using the bvn method"

}

ABT <- #The allelic test

function(data){

if((data[1,1]<0.5)||(data[1,2]<0.5)||(data[1,3]<0.5)||(data[2,1]<0.5)||(data[2,2]<0.5)||(data[2,3]<0.5)){

data=data+matrix(rep(0.5,6),nrow=2)

}

rr=data[1,]

ss=data[2,]

nn=apply(data,2,sum)

r=sum(rr)

s=sum(ss)

n=sum(nn)

pd=(2*rr[3]+rr[2])/(2*r)

ph=(2*ss[3]+ss[2])/(2*s)

p=(2*nn[3]+nn[2])/(2*n)

u=pd-ph

v=p*(1-p)*(1/(2*r)+1/(2*s))

re1=u/sqrt(v)

re2=1-pchisq(re1^2,df=1)

if(re2>=0.05){

re3="null hypothesis: association doesn't exist under significant level 0.05"

}

if(re2<0.05){

re3="alternative hypothesis: association exists under significant level 0.05"

}
