# Supplementary material for: Genome-wide association testing in malaria studies in the presence of overdominance
Source: Malar J. 2023 Apr 10;22:119. doi: 10.1186/s12936-023-04533-2 (PMC10084622; doi:10.1186/s12936-023-04533-2)
Supplement: Supplementary file 8 — Additional file 8: Text S3. Manhattan and quantile-quantile plots for selected chromosomes for Kenyan datasets. [file 12936_2023_4533_MOESM8_ESM.docx]

Additional File 8: Text S3: Manhattan and quantile-quantile plots for selected chromosomes for Kenyan datasets

Manhattan and the quantile-quantile plots for selected chromosomes were conducted for the Kenyan datasets using the *qqman* package [7].

setwd("D:/tests - 904")

Data1=read.csv("data_ch1max.csv")

Data2=read.csv("data_ch2max.csv")

Data3=read.csv("data_ch3max.csv")

Data4=read.csv("data_ch4max.csv")

Data5=read.csv("data_ch5max.csv")

Data6=read.csv("data_ch6max.csv")

Data7=read.csv("data_ch7max.csv")

Data8=read.csv("data_ch8max.csv")

Data9=read.csv("data_ch9max.csv")

Data10=read.csv("data_ch10max.csv")

Data11=read.csv("data_ch11max.csv")

Data12=read.csv("data_ch12max.csv")

Data13=read.csv("data_ch13max.csv")

Data14=read.csv("data_ch14max.csv")

Data15=read.csv("data_ch15max.csv")

Data16=read.csv("data_ch16max.csv")

Data17=read.csv("data_ch17max.csv")

Data18=read.csv("data_ch18max.csv")

Data19=read.csv("data_ch19max.csv")

Data20=read.csv("data_ch20max.csv")

library(qqman)

par(mfrow=c(3,3))

manhattan(subset(Data1, CHR == 1), highlight = snpsOfInterest, xlim = c(0, 50000000), ,ylim=c(0,20), main = "Chr 1")

manhattan(subset(Data2, CHR == 2), highlight = snpsOfInterest, xlim = c(0, 50000000), ,ylim=c(0,20), main = "Chr 2")

manhattan(subset(Data3, CHR == 3), highlight = snpsOfInterest, xlim = c(0, 50000000), ,ylim=c(0,20), main = "Chr 3")

manhattan(subset(Data4, CHR == 4), highlight = snpsOfInterest, xlim = c(0, 50000000), ,ylim=c(0,20), main = "Chr 4")

manhattan(subset(Data5, CHR == 5), highlight = snpsOfInterest, xlim = c(0, 50000000), ,ylim=c(0,20), main = "Chr 5")

manhattan(subset(Data6, CHR == 6), highlight = snpsOfInterest, xlim = c(0, 50000000), ,ylim=c(0,20), main = "Chr 6")

manhattan(subset(Data7, CHR == 7), highlight = snpsOfInterest, xlim = c(0, 50000000), ,ylim=c(0,20), main = "Chr 7")

manhattan(subset(Data8, CHR == 8), highlight = snpsOfInterest, xlim = c(0, 50000000), ,ylim=c(0,20), main = "Chr 8")

manhattan(subset(Data9, CHR == 9), highlight = snpsOfInterest, xlim = c(0, 50000000), ,ylim=c(0,20), main = "Chr 9")

manhattan(subset(Data10, CHR == 10), highlight = snpsOfInterest, xlim = c(0, 50000000), ,ylim=c(0,20), main = "Chr 10")

manhattan(subset(Data11, CHR == 11), highlight = snpsOfInterest, xlim = c(0, 50000000), ,ylim=c(0,20), main = "Chr 11")

manhattan(subset(Data12, CHR == 12), highlight = snpsOfInterest, xlim = c(0, 50000000), ,ylim=c(0,20), main = "Chr 12")

manhattan(subset(Data13, CHR == 13), highlight = snpsOfInterest, xlim = c(0, 50000000), ,ylim=c(0,20), main = "Chr 13")

manhattan(subset(Data14, CHR == 14), highlight = snpsOfInterest, xlim = c(0, 50000000), ,ylim=c(0,20), main = "Chr 14")

manhattan(subset(Data15, CHR == 15), highlight = snpsOfInterest, xlim = c(0, 50000000), ,ylim=c(0,20), main = "Chr 15")

manhattan(subset(Data16, CHR == 16), highlight = snpsOfInterest, xlim = c(0, 50000000), ,ylim=c(0,20), main = "Chr 16")

manhattan(subset(Data17, CHR == 17), highlight = snpsOfInterest, xlim = c(0, 50000000), ,ylim=c(0,20), main = "Chr 17")

manhattan(subset(Data18, CHR == 18), highlight = snpsOfInterest, xlim = c(0, 50000000), ,ylim=c(0,20), main = "Chr 18")

manhattan(subset(Data19, CHR == 19), highlight = snpsOfInterest, xlim = c(0, 50000000), ,ylim=c(0,20), main = "Chr 19")

manhattan(subset(Data20, CHR == 20), highlight = snpsOfInterest, xlim = c(0, 50000000), ,ylim=c(0,20), main = "Chr 20")

par(mfrow=c(3,3))

qq(Data1$P, main = "Chr 1")

qq(Data2$P, main = "Chr 2")

qq(Data3$P, main = "Chr 3")

qq(Data4$P, main = "Chr 4")

qq(Data5$P, main = "Chr 5")

qq(Data6$P, main = "Chr 6")

qq(Data7$P, main = "Chr 7")

qq(Data8$P, main = "Chr 8")

qq(Data9$P, main = "Chr 9")

qq(Data10$P, main = "Chr 10")

qq(Data11$P, main = "Chr 11")

qq(Data12$P, main = "Chr 12")

qq(Data13$P, main = "Chr 13")

qq(Data14$P, main = "Chr 14")

qq(Data15$P, main = "Chr 15")

qq(Data16$P, main = "Chr 16")

qq(Data17$P, main = "Chr 17")

qq(Data18$P, main = "Chr 18")
