# Supplementary material for: Genome-wide association testing in malaria studies in the presence of overdominance
Source: Malar J. 2023 Apr 10;22:119. doi: 10.1186/s12936-023-04533-2 (PMC10084622; doi:10.1186/s12936-023-04533-2)
Supplement: Supplementary file 9 — Additional file 9: Text S4. Simulation codes. [file 12936_2023_4533_MOESM9_ESM.docx]

Additional File 9: Text S4 Simulation codes.

#Note: we have to transpose the vectors to have data in the correct 2 by 3 table format

#different number of cases and controls for each SNP (total to be 200-5000)

#10 snps at a time, same format as MalariaGEN data

prob=c(0.25,0.5,0.25)

data=matrix(data=NA,nrow=10,ncol=6)

for(i in (1:nrow(data))){

n=sample(200:5000)

n1=sample(200:n)

n2=n-n1

cases=t(rmultinom(1,n1,prob))

controls=t(rmultinom(1,n2,prob))

data[i,]=cbind(cases,controls)

}

data=data.frame(SNP=c(1:nrow(data)),data)

data

#Renaming columns to match MalariaGEN data

names(data)[2]='cases_AA'

names(data)[3]='cases_AB'

names(data)[4]='cases_BB'

names(data)[5]='controls_AA'

names(data)[6]='controls_AB'

names(data)[7]='controls_BB'

data
