# Supplementary material for: Genome-wide association testing in malaria studies in the presence of overdominance
Source: Malar J. 2023 Apr 10;22:119. doi: 10.1186/s12936-023-04533-2 (PMC10084622; doi:10.1186/s12936-023-04533-2)

Additional File 10: Figure S1: Manhattan plots for additive, dominant, recessive and overdominant models for selected chromosomes. MAX test performs model selection using the *P-*value approach for Kenyan datasets.

The blue line indicates the threshold used in our study (5*10^-5) while the red line indicates the global threshold (5*10^-8).


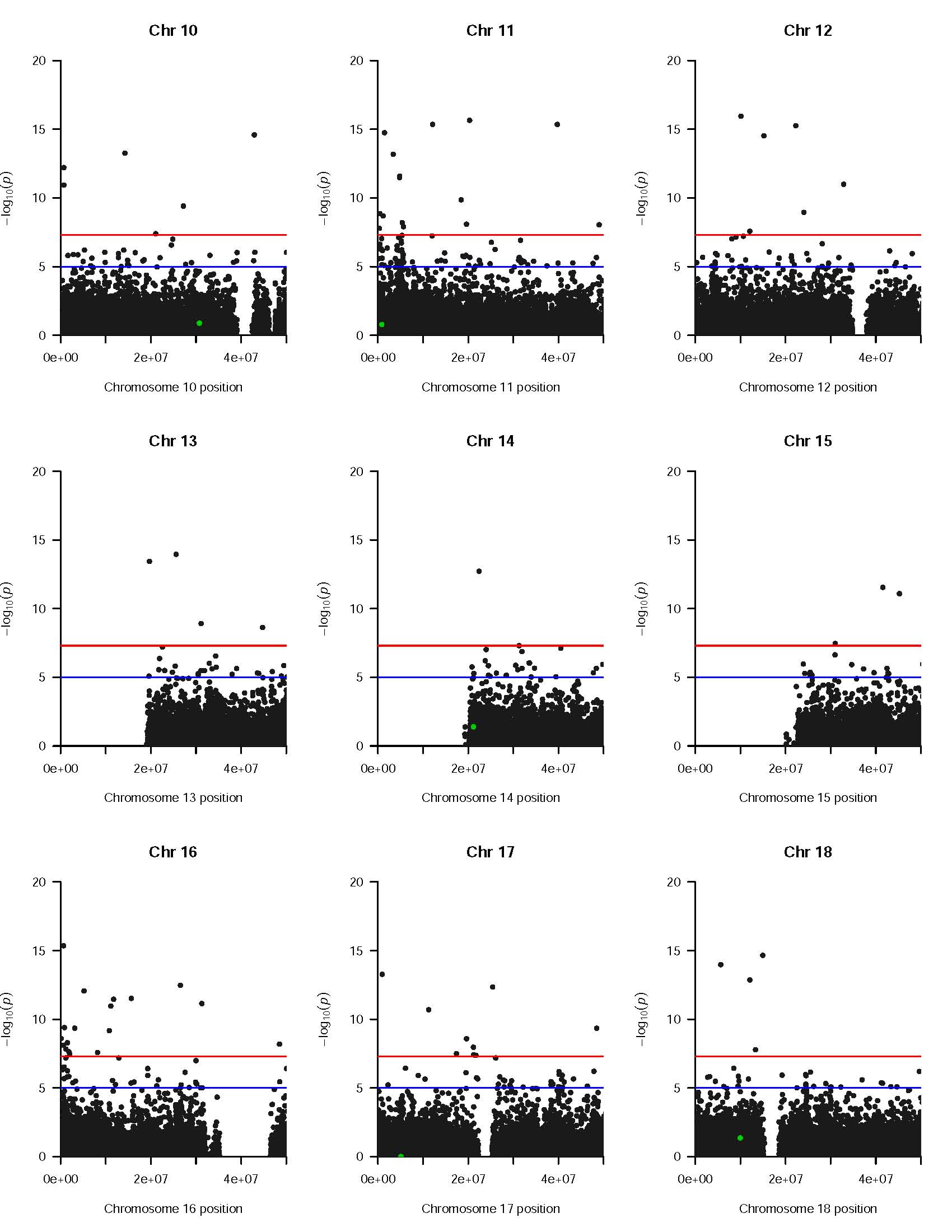

Supplement: Supplementary file 10 — Additional file 10: Figure S1. Manhattan plots of association findings for additive, dominant, recessive, and overdominant models for selected chromosomes. The MAX4 test performs model selection using the P-value approach for the Kenyan datasets. [file 12936_2023_4533_MOESM10_ESM.docx]
