# Supplementary material for: Genome-wide association testing in malaria studies in the presence of overdominance
Source: Malar J. 2023 Apr 10;22:119. doi: 10.1186/s12936-023-04533-2 (PMC10084622; doi:10.1186/s12936-023-04533-2)
Supplement: Supplementary file 11 — Additional file 11: Figure S2. Quantile-quantile plots of association findings for additive, dominant, recessive, and overdominant models for selected chromosomes. The MAX test performs model selection using the P-value approach for the Kenyan datasets. [file 12936_2023_4533_MOESM11_ESM.docx]

Additional file 11 Figure S2: Quantile-quantile plots for additive, dominant , recessive and overdominant models for selected chromosomes. The MAX test performs model selection using the P-value approach for Kenyan datasets.

The deviation from the red line, the expected frequency, is an indication of genetic association of malaria with SNPs in the genotyped regions.


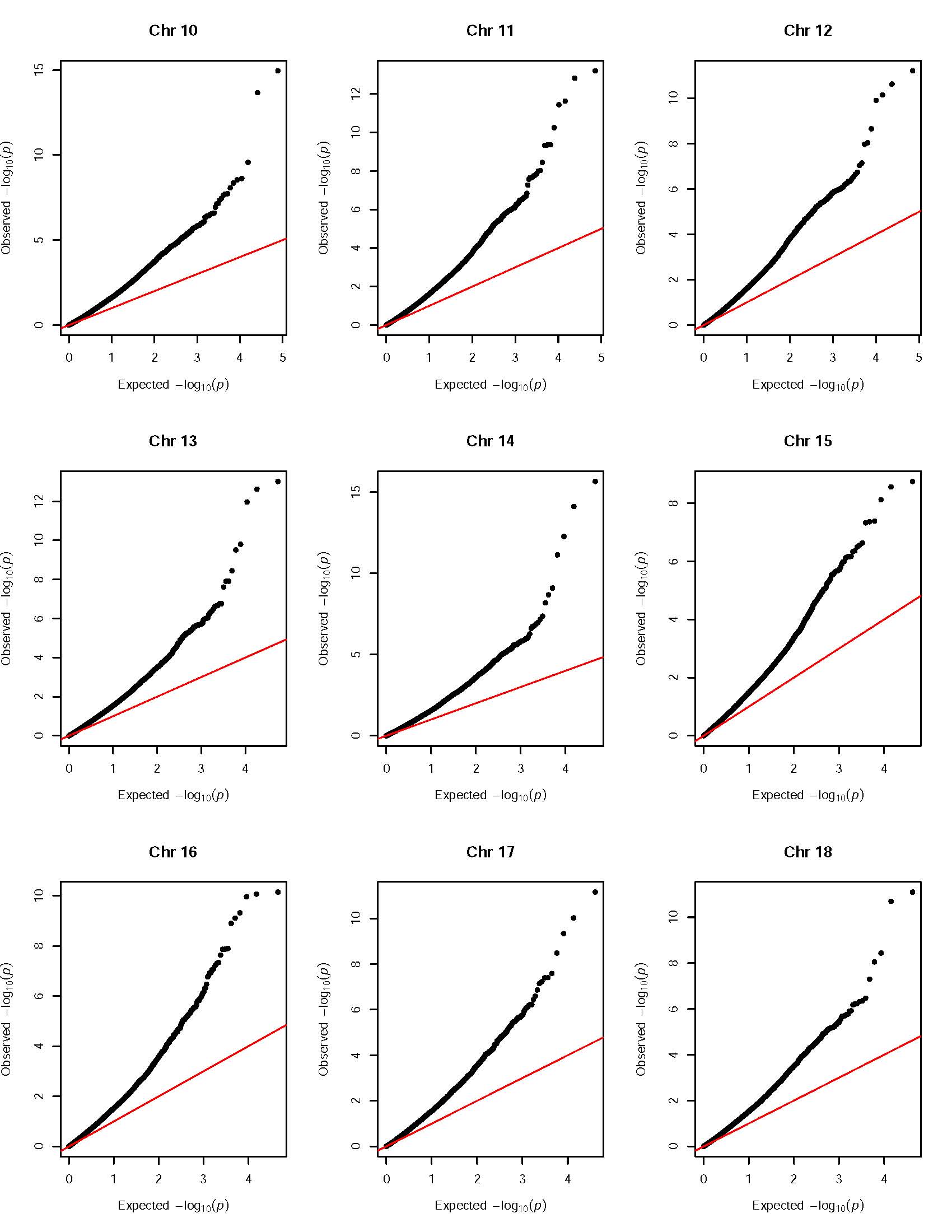


*Reference*

[1]Klein, R.J., Zeiss, C., Chew, E.Y., Tsai, J.-Y., Sackler, R.S., Haynes, C., Henning, A.K., SanGiovanni, J.P., Mane, S.M., Mayne, S.T., et al.: Complement factor h polymorphism in age-related macular degeneration. Science 308(5720), 385–389 (2005)

[2]Klein, R.J., Zeiss, C., Chew, E.Y., Tsai, J.-Y., Sackler, R.S., Haynes, C., Henning, A.K., SanGiovanni, J.P., Mane, S.M., Mayne, S.T., et al.: Complement factor h polymorphism in age-related macular degeneration. Science 308(5720), 385–389 (2005)

[3]Hunter, D.J., Kraft, P., Jacobs, K.B., Cox, D.G., Yeager, M., Hankinson, S.E., Wacholder, S., Wang, Z., Welch, R., Hutchinson, A., et al.: A genome-wide association study identifies alleles in fgfr2 associated with risk of sporadic postmenopausal breast cancer. Nature genetics 39(7), 870–874 (2007) 17.

[4]Consortium, W.T.C.C., et al.: Genome-wide association study of 14,000 cases of seven common diseases and 3,000 shared controls. Nature 447(7145), 661 (2007)

[5].R Core Team: R: A Language and Environment for Statistical Computing. R Foundation for Statistical Computing, Vienna, Austria (2021). R Foundation for Statistical Computing. https://www.R-project.org/

[6]Zang, Y., Fung, W. K., & Zheng, G. (2010). Simple algorithms to calculate asymptotic null distributions of robust tests in case-control genetic association studies in R. *Journal of Statistical software*, *33*, 1-24.

[7]Turner, S. D. (2014). qqman: an R package for visualizing GWAS results using QQ and manhattan plots. *Biorxiv*, 005165.
